# Southern Medical and Surgical Journal

**Published:** 1845-01

**Authors:** 


SOUTHERN MEDICAL AND SURGICAL JOURNAL.

This periodical, emanating from the Medical College of Georgia, having been suspended at the death of its editor, Professor Anthony, has been revived, and the first number of the new series is before us, in a good looking pamphlet of 48 pages. The work is under the editorial management of two members of the faculty of the Medical College of Georgia, Paul F. Eve, M.D., and I. P. Garvin, M.D. The first number contains a fair proportion of original matter, and the articles appear to have been written with care and ablity.

This makes the third medical journal which has been *enterprised* in the South and West in the last year—the Illinois Medical and Surgical Journal, the New Orleans Medical Journal, and the one which stands at the head of this article. They are none too many, for the readers are plenty in all the region over which they are meant to circulate. If they can secure a good share of the patronage which ought to be extended to journals of medicine, their success will be no longer doubtful. Y.

